# Artistic Dentistry

**Published:** 1852-01

**Authors:** A. Hill


From the New York Dental Recorder.

### ARTISTIC DENTISTRY.

*An Essay read before the American Society of Dental Surgeons, in their Twelfth Annual Meeting, held at Philadelphia, August* 5, 1851.—By A. Hill, D. D. S.

Gentlemen of the American Society—The subject of the present paper I have entitled *Artistic Dentistry.* My object has been to draw out before the mind more distinctly certain topics not generally noticed with as much particularity as the subjects seemed to demand.

It has occurred to me, that this department of our profession has not received that attention, from those who have written upon dental subjects, to which it is entitled.

Many able pens have been employed to much advantage in setting forth, in all their minuteness of detail, the different *mechanical* contrivances and the various modes of their application, so far as they are considered essentially useful to the practitioner of Dental Surgery.

Anatomy and Physiology, Chemistry and Materia Medica, with general Therapeutics, have each contributed, with a good degree of liberality, their respective quotas to ennoble and enrich this useful profession.

But I respectfully submit, that neither the skill of the accomplished surgeon, nor the ingenuity of the industrious mechanic, is adequate to the full accomplishment of that which is aimed at by the members of the dental profession, or demanded by the wants of those who require their services.

The physician may exhaust his skill in the treatment of disease; the surgeon, as a last resort, may be required to amputate a limb, or remove a dangerous cancer, and acquit himself perfectly in the dexterous use of the *knife*, the *saw*, and the *bandage;* and as the poor patient recovers, minus one or more of his limbs, his services are no longer requisite—his legitimate work is done.

The artistic skill of one who has an eye to the beauty of *form* and *proportion*, may now be summoned, who shall mould a limb and form a foot with so much excellence as to compensate, to some extent for the loss, and almost rival nature herself, in its beautiful adaptations.

But not so with the dentist. It is demanded and expected of him that he fulfil the several and distinct functions of the physician and surgeon, the mechanic and artizan.

He must not merely treat diseases of the mouth and teeth, in all their various relationships and multiform sympathetic connections, but he must extract, clean, regulate and restore, file and fill, and thus necessarily invade the domain of the surgeon, mechanic and artist.

What wonder is it, that comparatively so few are competent to meet and fulfil the public expectation in these various particulars.

And how sadly mistaken is he who supposes that a few weeks, or months at most, are sufficient to qualify himself for the duties of such a profession.

In strict language, surgery may be defined to be "the act of healing by manual operation."

Art, as applied to our profession, may be considered distinctively, as in those cases where the mind or the imagination is chiefly concerned, and may not improperly be classified with the more liberal, polite, and elegant arts of *poetry*, *music*, and *painting*.

Emotions, as distinct as the prismatic colors of the rainbow, are known to supply the necessary inspiration in each of these several departments; and yet they may become blended in such a delightful manner as to leave no distinct and perceptible trace of the precise line of their union.

The difference between the mechanic and the poet, the musician and the painter, can only be properly comprehended by a careful analysis of each.

It will, however, be sufficient for our present purpose to take one or two illustrations, by which these characteristic marks of distinction may be seen, and rendered applicable to the subject we have taken in hand.

The eye of an ingenious mechanic may sweep over a beautiful landscape, embracing

> "Upland grove and dell",—or
> Mountain, valley and river.

But how entirely different are his emotions from those of the genuine *poet*, *musician*, or *artist*. Observe them for a moment. You will see

> "The poet's eye, with a fine phrenzy rolling."

You will see his soul displaying itself over every lineament of his face, while he drinks in the delicious inspiration of the scene. The musician hears the dim

> "Music of the spheres"—

The babbling music of the brawling brooks, the choral anthems of the feathered songsters, until his wrapped soul can be silent no longer. Meantime the artist is reveling in all the beautiful combinations of light and shade—the sunlight that mildly quivers on the mountain-top, and streams to softest beauty down into the valley below. And those elegant tints of multiform foliage display themselves before his vision, until he instinctively seizes the pencil and transfers them to his canvas.

How various—yet how distinct, these impressions!

The poet breaks out in a rhapsody—the musician sweeps his lyre—the artist is seated at his easel—while the mechanic is coolly and tamely querying within himself as to "what tree will make the best shingles," and how the timber, which he sees before him, can be best made to subserve the purposes of his calling.

And these characteristic marks of distinction are not altogether peculiar to those individuals which we have named, but are a frequent matter of observation among all classes of men, and in every department of industry.

The peculiarity of which we speak, constitutes the difference between the coarse blunt form, rude spars and unwielding movements of an old Dutch ship, or a Chinese junk, and the elegant clipper-built vessels of the present day. The former resembling nothing but the most ungainly things, either on sea or land, and would almost puzzle a stranger to tell whether they were ever intended for nautical purposes or not. While the latter, so beautiful in their proportions, so symetrical in their arrangements, and perfectly adapted to the purposes for which they are designed, may justly be said to "Walk the water like a thing of life."

It is the display of artistic talent, in combination with high attainments in the mechanic arts, that constitutes this difference, not merely in the cases we have cited, but in every department of genius and industry.

Having briefly called your attention to the subject, with a few illustrations designed to show more distinctly the peculiar topic we have chosen as the subject of the present paper, we will now proceed to point out its more pertinent application to our profession.

In the manufacture, adjustment, and adaptation of artificial teeth and their appendages, this principle finds its fullest scope. No other branch of our profession offers a field so inviting and ample as this, in which to display true *artistic* skill (as distinguished from pure mechanical and scientific attainments) as this department of dental operations.

It is absolutely indispensable here from first to last. From the *moulding* and *carving* of the clay itself, to the *painting*, *glazing* and setting, *artistic skill* alone can ensure success.

The carving of mineral blocks is a species of sculpture where the truest and most clever artist cannot fail of distinction. Even Powers himself, were he to employ his genius upon a matter of this kind, would, I doubt not, exhibit the same masterly hand that has so exquisitely moulded the inimitable statue of the Greek Slave. And that which distinguishes one piece of this kind from another, is the same thing that distinguishes all his chaste and beautiful productions from the inferior specimens of those who would fain emulate his unrivalled excellence. And it would be almost as proper to compare some of those specimens which we sometimes see upon tomp-stones in some of our country grave-yards, with these gems of art, as to institute a comparison between the productions of some who are laboring in this department of the dental profession. Great strength some of them most undoubtedly have, and so have our New England ox-carts; and they will "stand the fire," and so will the "Salamander safes." "They will do for grinding," so will mill-stones: and if these qualifications (which, by the way, are very essential) are the only ones to recommend them, the day has gone by when they will be very likely to find a market.

But it affords me great pleasure to know that there are those in our own country, who cater to the wants of the community in this regard, who have no need to be ashamed of their productions. And some of them, *especially*, seem to have left but little more to be desired, in order to render an artificial denture all that the most sanguine aspirations of the profession have ever anticipated.

No specimens which I have seen, have served more to deepen this conviction upon my own mind, than some of those beautiful specimens from the laboratory of Jones, White & Co. Here is something besides those qualifications above referred to, as being common to the *ox-cart*, the *Salamander*, and the "upper and nether mill-stones."

Observe them, and you will see the delicate touches of the artist's pencil, displaying the skill of something more than a *mere* amateur in this business. Those delicate shades around the festooned borders of those *gum teeth* are such as, when properly mounted and adapted to the mouth, almost rival nature herself in her beautiful productions.

But I am happy to know that these gentlemen are not alone in running this race for distinction. Others are ably competing for this prize, and to whom the final award of superior excellence will be made, is certainly more than I am able to say.

Among others, whose elegant productions are worthy of being mentioned as a further illustrative of the peculiar feature we are seeking to develop, the names of such men as Stockton, Alcock, Morton, Crosby, and many more, probably, whose specimens we have never had the good fortune to see, should not be omitted.

We thus particularize, not by any means to seem invidious, but first, to illustrate this feature of our subject; and secondly, to award praise where it is unquestionably due.

The rapid strides of this rapidly advancing department of our useful profession have, for the last few years, been such as almost to pass the bounds of credibility.

This advancement, especially in *Artistic* Dentistry, may be very clearly seen by attending for a moment to the following extract from a work on Dental Science, published in 1834. The work is entitled, "A Treatise on the Anatomy and Physiology of the Teeth, &c.: their diseases and treatment, with practical observations on Artificial teeth, and rules for their construction. By David Wemyss Jobson, M. R. C. S. E., Dentist in ordinary to his Majesty, and to his Royal Highness the Duke of Sussex," &c.

Now, with such a flourish of trumpets in 1834, what do we find upon the subject of artificial teeth? The following extracts from his pen will show. He says: "Artificial teeth have also, of late years, been made from a *porcelaneous substance*, and under the name of 'mineral' and 'terro metallic' teeth, have afforded an extensive range for empirical deception. The attraction held out is their alleged 'incorruptibility,' by which term the unwary are entrapped and led to believe that teeth of this description are much more durable than the natural ones, *i. e.* natural teeth artificially used.

"The very reverse of this is the case. For although they are not subject to change of color, yet they are in every instance so brittle as to be easily broken off on coming in contact with those of the opposite jaw. * * * * * *

"When these mineral China teeth were first introduced into this country from France, (for it is to our neighbors on the opposite side of the channel that we owe these as well as many other similar ephemeral productions,) the greatest mystery was affected on the subject of their composition, although any of our potters, or porcelain makers, could easily have disclosed it. * * * * * * * * *

"The most extravagant expectations were then formed from them; although few, or rather none of the advantages which they were supposed to possess, have been realized, and they are now considered to be a complete failure. They have never been much used by the leading dentists of the day, and I believe are now wholly discountenanced by the respectable part of the profession, although they still reign paramount with the disreputable."

This extract, although somewhat lengthy, serves to show in a very clear light the progress of *artistic* dentistry (at least in this country) within the last ten or fifteen years. And I am thinking that if "Johnny Bull" has not pretty essentially waked up to this matter within this period, that some of those Yankee specimens, now to be seen in the great World's Fair, will very much surprise him. But how oddly these remarks sound to us, who are now so familiar with these really beautiful gems of the dental art.

In another part of this work, this same writer goes on to describe the process of staining artificial gums made of bone, ivory, and the teeth of the Hippopotamus, by immersing them into a boiling solution of cochineal, red saunders wood and vinegar.

These rude attempts at imitating nature's exquisite pencilings, although but the feeble beginnings of what we now see, yet, nevertheless, contain in them the hopeful prophecy of the glorious future, with respect to art, as applied to our profession.

While we take great pleasure in awarding high praise to those who, in our own day, have achieved so much, candor and truth compel us to say, that with respect to the formation and coloring of artificial gums, the goal is not yet reached. To a certain extent, one artist has copied another, when they should have been copying *nature most critically*. This we think must be obvious, where even the *best* specimens are placed in juxtaposition with the natural gums of a healthy mouth.

In calling attention to this subject in this particular manner, we trust we shall not be accused of the faintest desire to underrate in the least what has already been accomplished.

But if the moulding, or sculpture and painting of mineral teeth and gums require the exercise of *so much* artistic skill, in order fitly and justly to represent the handiwork of nature, it is only a small part of what we deem essential to their successful use and application.

It is in their adjustment and adaptation to the mouth and face, that real artistic skill, and scientific attainments combined, are more essentially necessary.

The error is quite common, not only among dentists, but throughout the community generally, that *any individual* possessing a tolerable share of mechanical ingenuity, is competent to do this business. But we think the absurdity and presumption of this thing will appear evident to every one who will attentively consider the following hints in their bearing upon the subject.

Every man has a *peculiar,* and in some respects, *distinctive* physiognomy. And these peculiarities constitute the unerring marks of personal identity. That which makes the individual look like himself and no one else. Now, no feature of the face can be changed or marred, either by accident or design, without imparting a different character to the physiognomy, and thus changing to a certain extent the relations of the individual. And hence the loss of an eye, or the involuntary contraction of a muscle, or the paralysis of the nerve of any muscle of the face, so that its natural play is impeded, may be and always are considered most unfortunate in their effects upon the physiognomy. But by no means more so than the loss or displacements of the dental organs. For here, the controlling influence of these organs are distinctly seen in all the physiognomical relations. The muscles of the cheeks, the moulding of the lips, the relation of the nose and chin, and that delightful play of the features that indicate happiness and pleasure.

In every age of the world, the "human face divine" has been a subject of interesting study and observation; and, to some extent, all men are physiognomists. Not that all men, like Lavater, or the phreno-physiognomists of the present day, are scientifically learned upon the subject; but *instinctively* and *practically*, they are and ever will be so. From the little child that intently gazes upon your countenance, either to be won by its welcoming smiles, or repulsed by its forbidding frown, to the man or woman of hoary hairs, all are practical physiognomists. And it must, we think, be acknowledged that any circumstance or combination of circumstances that are capable of changing the whole expression of the face, and of leaving upon the minds of our children and friends an impression so important and enduring, is not to be disregarded or overlooked. It is of the teeth and the features of the mouth, as related to physiognomy, that these remarks are to be applied.

It has not escaped the attention of close observers upon this subject, that every class of inferior animals, as well as distinct species of the human family, are characterized by the circumstances to which I have referred.

The researches of that great naturalist, Cuvier, has shown that the teeth alone are sufficient to determine the class of animals to which they belong, although the fossil remains accompanying them, may be otherwise deficient. But why is this, if the teeth are not strikingly characteristic?

The teeth of the Carnivora are at once striking and peculiar. The Graminivorous and Herbivorous are also distinctly marked. Man, who, strictly speaking, belongs to neither class exclusively, yet partakes, to some extent, of the peculiarities of each, and may, therefore, be said to be Omniverous; is, nevertheless, sometimes characterized by a striking resemblance to some one of these distinct classes. Indeed, this is so manifest in some instances, that these very circumstances will enable us to read the character of the individual possessing them with as much certainity and almost equal facility, that the Messrs. Fowlers can do it by the superfices of the cranium.

Who has not seen the distinctive marks of the lion, the mastiff, and the bull-dog, in the mouth, teeth and lips of some men? And the cow, the monkey, or the rat, as distinctly in others? And what difficulty have we in predicating certain traits of character, peculiar to that class of animals, of these distinctive signs?

For illustration, suppose we find in the mouth of any patient those sharp pointed teeth, peculiar to the carnivora, so strongly marked as to excite our special attention. I ask is there any risk in affirming of that individual certain traits of character which are known to distinguish that class of animals? Of course, these peculiarities are variously modified according to circumstances, but yet sufficiently marked for the purposes to which we refer. But if you will pardon this seeming digression, I would observe further, that those long, smooth square-edged teeth, peculiar to the *kine* or *herbiverous* class of animals, not only indicate a constitutional preference for a vegetable diet or regimen, but a docile temper and tractable disposition.

These facts (if such they are) are certainly not without their value, not merely in a speculative, but in a physiological point of view. They certainly ought to be considered by the physician whenever required to prescribe a rule of diet for any individual. And they seem to me to militate with no inconsiderable force against the exclusive notions of the so-called "vegetarians."

However interesting it might be to pursue this train of thought, it would make this paper far exceed its intended limits, if we should attempt its complete development. But we have called your attention to it mainly to show its important relation to the subject we have taken in hand.

We shall generally find that these peculiarities, where strongly marked, influence in a remarkable manner the whole physiognomy. The nose and lips, as well as maxillary bones, receive a peculiar conformation, adapted to the denture as it exists or as it once existed. And where the law of sympathy is supposed to act, the relations between the several parts must be established accordingly. Hence we find short, square, and generally strong and regular teeth in short, chubby, round-faced individuals. And so uniformly is this the case, that it ought never to be overlooked or disregarded by the dentist, whose duty it may be to supply an artificial denture. We should not be willing to assert that the tallest persons in the community *invariably* have the longest teeth. But we do say that, *veterus parabus*, this is generally the case.

It is sometimes true that very tall persons have very short faces and small heads. They may be in some instances, it is true, like certain tenements which you have seen in this particular—" Long between joints and low in the garret." In a case like this, you will doubtless find a great disproportion in the length of the teeth, as compared to the body and limbs. But these may be considered rather as exceptions to the rule, than as constituting the rule itself.

Nature is generally harmonious in matters of this kind ; and seldom places a short trunk and diminutive head upon long, slim pedestals. And the relative size of the body, its peculiar conformation, and other circumstances, may be told with as much precision and exactness from seeing the teeth alone, as it is possible to infer the same from any other detached portion. The law of relative proportion is such, that these circumstances seem to us indubitable.

The following extract, from a recent scientific work, is so full of interest, and so much to the point under consideration, that I trust I shall be justified in quoting it here. It is as follows:

" In the extensive quarries of gypsum, near Paris, the workmen frequently dug out the bones of unknown animals. In the mind of the uneducated laborer, they excited little attention, and were thrown away.

" The fact, however, coming to the knowledge of Cuvier, he undertook to examine the matter. The result of the examination astonished himself and the world. He discovered upwards of fifty species of animals not known on earth. They all belonged to the order which naturalists call the *pachydermata*, or thick skinned, of which the horse, the hog, and the rhinoceros are examples. They were not, however, the horses, hogs, or anything else now living on the globe.

"Cuvier's own account of the manner in which he succeeded in re-constructing these strange skeletons, is peculiarly interesting. I found myself (says he) as if placed in an immense charnel-house, surrounded by mutilated fragments of many hundred skeletons, of more than fifty kinds of animals, piled confusedly around me. The task assigned me was to restore them all to their original position. At the voice of comparative anatomy, every bone and fragment of bone resumed its place. I cannot find words to express the pleasure I experienced in seeing, as I discovered one character, how all the consequences which I had predicted from it were confirmed. *The feet were found in accordance with the character announced by the teeth; the teeth in harmony with those indicated beforehand by the feet;* the bones of the legs and thighs, and every portion of the extremities, were found set together precisely as I had arranged them before my conjectures were verified by the discovery of the parts entire. In short, each species was, as it were, re-constructed from a *single one* of its component elements."

Now, if these considerations are entitled to any weight, as tending to throw light upon the uniformity, consistency and harmony of nature's own works, their pertinency to the subject before us must be seen at once. And something more than mere mechanical skill is required in preparing and adjusting artificial teeth.

If it be true that the size, shape, color and position of the teeth in man, conveys to the mind of the beholder certain distinct impressions as to *character* and *temperament*, then it is easy to be understood how the dentist may *libel a man, and destroy his character completely.*

If he puts in the mouth of a broad-shouldered, thick-necked, chubby, laughing-faced, individual, an entire set of long, slender, and delicately transparent, or pearly teeth, he has outraged nature in her own temple, and caricatured his confiding victim. Such teeth as these do not belong to this variety of the species, any more than the teeth of an insignificant poodle belongs to a mastiff or a brave Newfoundland dog. And the varieties in the former are as remarkable as in the latter case.

Equally unbecoming and improper would it be to substitute large, short yellow teeth in the mouth of a delicate young lady of a nervo-sanguine temperament, blue eyes, clear complexion, and a pulmonary diathesis.*

Age, too, has its distinctive marks. Not that we are to examine the mouth of men and women, as they do horses, to learn how old they are; not so. But I contend that the operator should be able to tell, from the numerous physical signs connected with this subject, the relative size, color and form of the teeth associated with the several varieties of temperament, form, figure and age of each individual.

In old age, the complexion changes—becoming more sallow and less transparent. And the same change as to color takes place in the teeth of aged persons.

The diminished force of the heart and arteries, the consequent moderation in the action of the capillary vessels, together with the corrugated or wrinkled condition of the skin in the case of aged persons, all tend to corroborate this statement, and may be regarded as so many signs hung out for our guidance in the pursuit of scientific knowledge.

The antagonism of the teeth is another point demanding our attention. Every one knows the effect upon the physiognomy which that peculiar protrusion of the under-jaw, known as "*jimber-jaw*," produces.

The style of the countenance, and the "*tout ensemble*" of

---

*The above remarks, in relation to the peculiar kind of teeth which properly belong to persons of different constitutional temperament, are worthy of especial attention. A dentist should be able to tell from the temperament and diathesis of his patient, just the kind of teeth needed to restore the loss of features and expression. This he should be able to do if the teeth are out before he sees his patient; yet how often do we see every rule which nature has pointed out as necessary utterly disregarded, and this even when there are teeth remaining to guide in form and color. The remarks which follow in relation to the articulation are also worthy of regard. We frequently meet with full sets of teeth which have been inserted, and so badly articulated that neither in or out of the mouth can they be brought together right. We recently saw a set which was made with special reference to exhibition, which did not articulate by half an inch.—Ed.

the face becomes identified with this malformation, and receives its peculiar physiognomical character from it. And every similar departure from a legitimate and regular arrangement of the features is, in so far, a departure from the true standard of personal beauty.

In the arrangement of an artificial denture, as to the matter of antagonism, much of personal beauty and individual character depend. And I might also add, much of the comfort and real utility of the teeth themselves.

How much annoyance, vexation and weariness people are sometimes compelled to endure in consequence of a false antagonism of the teeth, may be known by attending to the following circumstances.

In the first place, nature has fixed the precise point of contact between these organs, and has adjusted the muscular movements of the parts accordingly. And it must be obvious that any alteration, involving the change of the leverage of the parts, must subject the muscles to a strain involving weariness and pain. And not only so, but every motion of the parts, either in eating or speaking, must be accommodated to the change.

This necessarily involves a comparative change in the arrangement of the features, and an expression of the countenance, at once unfortunate and unnatural. To adjust these parts in harmony with nature's own plan, so as to preserve the just balance of power, the easy and natural motion of the parts, and the regular and uniform play of the features, is absolutely indispensable to success, and is therefore most desirable.

Moreover, any variation from this point, even though it be but the twelfth or sixteenth part of an inch, necessarily changes the fulcrum, and diminishes the power and ease with which mastication is performed.

I have become satisfied, from observations upon this point, that too little attention has been given to these considerations by the members of our profession. And it has doubtless been observed by you all, in the course of your experience, that the teeth artificially supplied, are sometimes too short and sometimes too long, and thus, in both instances, imparting a false character to the person who wears them. And it is only by attending to some of the suggestions herein made, that a remedy can be supplied. A perfect imitation of nature is the perfection of art.

The very best productions of the greatest masters, in painting and statuary, always have and ever must derive their greatest excellence from the fidelity and skill with which they copy nature. Aside from this, neither painting nor sculpture possess any excellence or are of any value.

And this resemblance, which is so essential in works of this kind, necessarily involve the strictest attention and deepest thought. Circumstances are of consequence to them, which, to men in other professions, are of little account. And the same thing is true of him who would distinguish himself in the department of dental surgery to which we refer. The form, figure, size, temperament, and complexion, are all to be consulted, and order and harmony restored.

Where malformations and actual deformity exist, real artistic dentistry may do much in changing and bringing back the legitimate form and expression of the face. Indeed, it must be in circumstances like these, that the most noble and splendid achievments are to be sought. And the restive spirit of professional ambition can never be satisfied until the results, so briefly alluded to, and so imperfectly foreshadowed, are fully realized.

